# Risk of heavy metal ingestion from the consumption of two commercially valuable species of fish from the fresh and coastal waters of Ghana

**DOI:** 10.1371/journal.pone.0194682

**Published:** 2018-03-23

**Authors:** Francis Gbogbo, Anna Arthur-Yartel, Josephine A. Bondzie, Winfred-Peck Dorleku, Stephen Dadzie, Bethel Kwansa-Bentum, Julliet Ewool, Maxwell K. Billah, Angela M. Lamptey

**Affiliations:** 1 Department of Animal Biology and Conservation Science, University of Ghana, Legon, Accra, Ghana; 2 Department of Biochemistry, Cell and Molecular Biology, University of Ghana, Legon, Accra, Ghana; 3 Department of Marine and Fisheries Sciences, University of Ghana, Legon, Accra, Ghana; Chinese Academy of Sciences, CHINA

## Abstract

**Background:**

The need to evaluate the human health safety of fishery resources remain urgent in the mist of the ever-increasing fear of heavy metal toxicity from the consumption of Ghana’s fisheries resource, as a consequence of pollution from several anthropogenic activities including artisanal gold mining. Nevertheless, the bigeye grunt (*Brachydeuterus auritus*) and Bagrid catfish (*Chrysichthys nigrodigitatus*) remain commercially valuable fish species in West Africa and continue to attract high patronage.

**Method:**

Forty-five specimens each of *C*. *nigrodigitatus* and *B*. *auritus* collected from the Weija Dam and the Tema Fishing Habour in Ghana, between June and September 2016, were analysed for seven heavy metals using Atomic Absorption Spectrometry.

**Result:**

Lead and Cadmium were below detection in all samples while Cu was not detected in *B*. *auritus*. Levels of the remaining metals (mg kg^-1^) were below FAO/WHO maximum permissible limits in fish and occurred in the rank order Se (3.5) > Zn (2.34) > Cu (0.59) > As (0.37) > Hg (0.19) in *C*. *nigrodigitatus* and Se (2.97) > Zn (2.28) > Hg (0.31) > As (0.21) in *B*. *auritus*. Only As in *C*. *nigrodigitatus* recorded Estimated Weekly Intake (EWI) greater than FAO/WHO Provisional Tolerable Weekly Intake (PTWI). Also, As in *C*. *nigrodigitatus* and Hg in *B*. *auritus* had Targeted Hazard Quotient (THQ) greater than 1 for individuals consuming the fishes on daily basis and therefore, raising concerns. However, for both species of fish, cancer risk of As was 1 in 10,000,000,000 and modified Health Benefits values of Se (HBV_Se_) were positive indicating the health risks that might accompany Hg exposure would be negated. Since toxicity depends on the concentration and quantity of a pollutant consumed, safe maximum consumption rate of *C*. *nigrodigitatus* based on As concentrations was 0.21 mg per day and that of *B*. *auritus* was 0.058 mg per day for Hg. With an average of 0.227 kg fish per meal of an adult human, these translated into not more than 24 *C*. *nigrodigitatus* and nine (9) *B*. *auritus* meals in a month but because fish is consumed at 0.0685 kg per person per day in Ghana, these values respectively translates to 93 and 30 safe days of consumption per month.

**Conclusion:**

At the rate of 0.0685 kg fish per person per day that fish is consumed in Ghana, the consumption of the two species of fish in Ghana would essentially be of little or no consequence to consumers.

## Introduction

Heavy metal contamination is a serious environmental concern not only because of the direct toxic effect of metals on organisms but also, the indirect effects of the consumption of metal contaminated food. Heavy metals such as Pb, Hg, Cd and As are toxic to life forms because of their inference with cellular processes, the results of which have been linked to a number of pathological conditions including neurological disorders, kidney damage, skin damage, circulatory system problems, and increased risk of cancer [1, 2]. On the other hand, some other heavy metals such as Zn, Fe and Cu, which are essential for normal human cellular functions at specific range of cellular concentrations, can become toxic at elevated tissue concentrations, or cause deficiency disorders at below normal tissue concentrations [3]. Pollution of the aquatic environment with heavy metals is particularly of global public health concern because fishes which are located at the end of the aquatic food chain may bioaccumulate and biomagnify heavy metals to toxic levels for human consumption.

Heavy metal contamination of the aquatic environment is increasingly becoming common in many developing countries including Ghana, where this has been linked to several anthropogenic processes including artisanal gold mining [4,5], electronic waste processing [6, 7], industrial processes [8], domestic sewage discharges [9] and agricultural activities. The pollution and degradation of land and water bodies from artisanal gold mining, for instance, are so wide spread in Ghana that efforts to combat the menace have recently been boosted nationwide [10, 11]. While most of these activities and contaminations occur in the terrestrial environment, the metals are transferred to rivers and the sea through fluvial processes, predisposing both fresh and marine water fisheries to the risk of heavy metal accumulation [12, 13]. Not surprisingly, a higher level of Hg were detected among Ghanaians compared to some other nationals in a recent study [14].

Fisheries have received a special attention as a potential source of heavy metal contamination to humans because of the wide consumption of fisheries [4, 15, 16, 17]. In West Africa, the bigeye grunt (*Brachydeuterus auritus*) and Bagrid catfish (*Chrysichthys nigrodigitatus*) are two commercially important fish species that are widely consumed and continue to attract high patronage. *Brachydeuterus auritus* is a semi-pelagic carnivore that inhabits coastal waters of the Eastern Atlantic, ranging from Mauritania to Angola [18, 19, 20]. It is an extremely valuable component of West African fisheries accounting for over 5% of the total marine fish catch in Ghana [21, 22]. *Chrysichthys nigrodigitatus* on the other hand, is a demersal omnivore inhabiting the bottom of shallow lakes and rivers ranging from Senegal to Angola [23, 24]. It is a widely eaten fish in Ghana [25] and across the African continent [26].

Giving the commercial importance of these two species of fish and the proximity of their habitat to heavy metal pollution activities, it is imperative that their heavy metals concentrations are assessed to determine the wholesomeness of the fishes for human consumption. Previous studies on heavy metal contaminations of Ghanaian fishery resources have focused largely on toxic metals. Literature on the selenium content of Ghana’s fisheries resources, for instance, is essentially non-existing although it is generally known that the toxicity of mercury is dependent on the availability of the essential heavy metal selenium [27], Additionally, *C*. *nigrodigitatus* is freshwater species as opposed to the marine habitat of *B*. *auritus*, with anticipated difference in the extent of heavy metal contamination between the two habitats.

**Fig 1:**
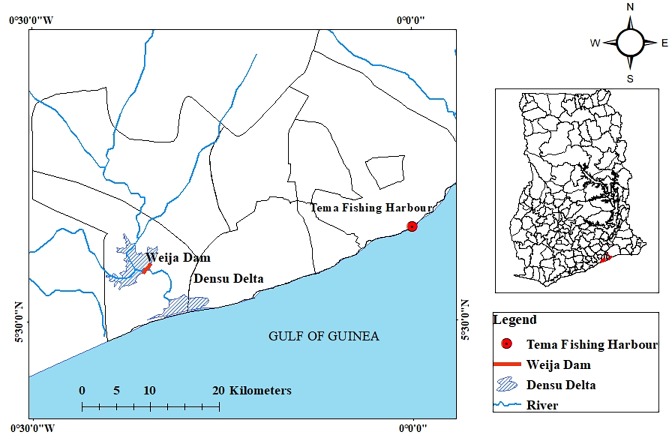
Map of Ghana showing the Weija Dam and the Tema fishing harbour where fish specimens were collected.

In this study, we assessed and compared the concentrations of mercury, arsenic, lead, cadmium, selenium, zinc and copper in *C*. *nigrodigitatus* and *B*. *auritus* landed in 2016 from the Weija Dam on the Densu River and the Tema Fishing Habour respectively, in Ghana (Fig 1). Further, we assessed the level of human health risk associated with the consumption of both species of fish by determining the Estimated Daily Intake (EDI), Targeted Hazard Quotient (THQ), modified Health Benefits Values of Se (HBV_Se_) and Cancer Risk of Arsenic. We further evaluated the relationship between the sizes and weight of the fishes with heavy metal concentrations and determined the maximum allowable fish consumption rates as well as the maximum number of fish meals that can be safely consumed over a period of a month. We predict that the concentration of heavy metals in *C*. *nigrodigitatus* would generally be higher than *B*. *auritus* due to the association of *C*. *nigrodigitatus* with the freshwater environment that is closer to the anthropogenic sources of heavy metal contamination as opposed to the marine habitat of *B*. *auritus* that are generally distant from the pollution sources with wider dilution effects. Consequently, we proposed that the consumption of *C*. *nigrodigitatus* would be associated with a higher human health risk than that of *B*. *auritus*. The significance of this information to the food safety and security can generally not be overemphasized.

## Materials and methods

### Sampling and metal determination

In the months of June, July and September 2016, specimens of *C*. *nigrodigitatus* and *B*. *auritus* were purchased onsite from local fishermen. The *C*. *nigrodigitatus* specimens were purchased from fishermen operating in the Weija Dam on the Densu River (Fig 1) while *Brachydeuterus auritus* specimens were purchased upon landing from the sea at the Tema Fishing Harbour (Fig 1). The Densu River is considered one of the most polluted rivers in Ghana as it receives untreated sewage from the several towns it traverses [5]. It is a noted area for high use of agrochemicals and harmful chemicals in fishing. On the contrary, fishing vessels landing at the Tema fishing harbour generally fish in the coastal waters of Ghana where pollutants including heavy metals from the catchment of many rivers end up through fluvial processes.

All specimens were purchased prior to sorting and for each species, 15 individuals were randomly selected from the harvested samples in each of the three months giving a total of 45 individuals per species and 90 specimens in all. The fish specimens were placed on ice and transported to the laboratory where their standard length and weight were measured. The specimens were then cleaned with double distilled water and 0.5 g of the skeletal muscle beneath the dorsal fin of each individual weighed into an acid cleaned vial and frozen at -20°C. Materials used in cutting the muscles including the forceps and scalpel were washed in dilute nitric acid. The muscle tissues (0.5 g) were digested in concentrated nitric acid and hydrogen peroxide (BDH Laboratory Supplies, Poole England), homogenized and topped to 100 ml after which aliquots were analyzed for As, Hg, Pb, Cd, Se, Zn and Cu using Varian AA240FS Fast Sequential Atomic Spectrometer. Standard Reference Material IAEA -350 (Tuna Fish homogenate) was analyzed to check precision of the method. Mean recovery (± Standard Deviation, n = 7) was 98 (±2.7)% for As, 99 (± 4.3)% for Hg, 97 (± 2.6)% for Pb, 101 (± 3.2)% for Cd, 101 (±3.3)% for Se, 99 (±4.5)% for Zn and 98 (± 3.7)% for Cu. Calibration was done with AA-standards and procedural blanks analyzed to check contaminations during sample preparations. Additionally, repeats were regularly carried out to check reproducibility. Concentrations were reported as an average of seven instrumental readings per unit wet weight.

### Data analysis

#### Metal concentrations and frequency distributions

Metal concentration data were tested for normality using Kolmogorov–Smirnov test. As data were not normally distributed, metal concentrations between *C*. *nigrodigitatus* and *B*. *auritus* were compared using Mann–Whitney U test. Within species comparison of the concentrations of the metals was also performed for each species using Kruskal–Wallis test. In processing data, values below detection limit (0.2 mg kg^-1^ for As, Pb, and Hg, 0.4 mg kg^-1^ for Cd, 0.6 mg kg^-1^ for Cu) were converted to fill values by dividing the limit of detection by the square root of 2. Spearman’s rank correlation analysis of metal concentrations with weight and standard length of fish were evaluated. Distribution frequency of the metal concentrations were compared to permissible limits of each metal in fish which was 0.5 mg kg^-1^ for Hg [28, 29, 30], 2 mg kg^-1^ for As [31], 50 mg kg^-1^ for Zn [32, 33, 34] and 40 mg kg^-1^ for Cu [32, 33, 34]. Because there is currently no permissible limit for Se in fish, we compared Se concentrations to the standard of 2.0 mg kg^-1^ reported by [35].

#### Estimation of Daily Intake

Estimated Daily Intake (EDI) of metals from the consumption of the two species of fish was determined according to Eq 1

$$EDI=(MC\;x\;DI)\;x\;BW{}^{-1}$$ (1)

Where MC, DI and BW respectively denote the heavy metal concentration in fish (mg kg^-1^ on wet weight basis), daily average intake rate of fish (68.5 g per person per day [36, 37] and average body weight (considered to be 70 kg per adult). Estimated Daily Intake of Zn and Cu were compared to FAO/WHO Provisional Maximum Tolerable Daily Intake (PMTDI) [5, 29,30] where PMTDI was 0.8 mg kg^-1^ bodyweight for Zn and 0.5 mg kg^-1^ bodyweight for Cu [32, 33]. Similarly, EDI of Hg and As were converted to Estimated Weekly Intake (EWI) by multiplying the EDI by 7 and then compared to FAO/WHO Provisional Tolerable Weekly Intake (PTWI) where PTWI was 4 μg kg^−1^ body weight for Hg and 2.1 μg kg^-1^ bodyweight for As [29]. In order to integrate selenium-specific nutritional benefits in relation to potential mercury exposure risks associated with the consumption of the two species of fish, the modified Se Health Benefits Values (HBV_Se_) for *C*. *nigrodigitatus* and *B*. *auritus* were determined according to Eq 2

$$HBVSe=\left(\frac{\left[Se-Hg\right]}{Se}\right)\times (Se+Hg)$$ (2)

Negative HBV_Se_ values indicate Se concentrations will not offer protection from Hg toxicity while positive HBV_Se_ values imply the health risks that might accompany Hg exposures would be negated by Se concentrations [38].

#### Estimation of Targeted Hazard Quotient

The non-carcinogenic health risks from consumption of the two species of fish by adults were assessed based on the Target Hazard Quotient (THQ) according to Eq 3.

$$THQ=(EF\times ED\times DI\times MC)\;x\;(RfD\times BW\times AT){}^{-1}$$ (3)

Where EF denotes exposure frequency (days per year), ED is the exposure duration (equivalent to average lifetime of 64 years for Ghanaian population) [39] RfD is the oral reference dose (As = 3 x 10^−4^ mg kg^-1^ day^-1^, Hg = 3 x 10^−4^ mg kg^-1^ day^-1^, Cu = 4.0 x 10^−2^ mg kg^-1^ day^-1^, Zn = 3.0 x 10^−1^ mg kg^-1^ day^-1^, Se = 5.0 x 10^−3^ mg kg^-1^ day^-1^ [40] and AT the average time of exposure to the chemical (365 days per year x ED). THQ was separately calculated at EF of 365 days per year for people who eat fish seven times in a week and 52 days per year for those who eat fish once a week. THQ values below 1 implies that the exposed population is unlikely to experience obvious adverse effects [41, 42]

#### Cancer Risk of Arsenic

Since inorganic arsenic is listed by USEPA as human carcinogen [43, 44], the lifetime Cancer Risk (CR) due to the consumption of As in the two species of fish was estimated according to Eq 4.

$$CR=(EF\times ED\times DI\times MC\times CSF)\;x\;(BW\times AT){}^{-1}$$ (4)

Where CFS denotes oral carcinogenic slope factor from the USEPA’s Integrated Risk Information System (IRIS) for inorganic As (1.5 mg kg^-1^ per day) [45]. As the proportion of inorganic arsenic in fish varies from 1 to 10% of the total arsenic [46], in the current study, we assumed inorganic arsenic to be 3% of the total arsenic concentration as suggested by [47, 48, 49]. According to the USEPA, acceptable lifetime cancer risk levels range from 10^−4^ (indicating a probability of 1 chance in 10,000 of an individual developing cancer) to 10^−6^ (indicating a probability of 1 chance in 1,000,000 of an individual developing cancer).

#### Maximum allowable consumption rate

Since the toxicity of a pollutant depends on its concentration and the quantity that is consumed, the maximum allowable daily fish consumption rate (CR*lim*) for non-carcinogenic heavy metals was calculated according to Eq 5.

$$CRlim=(RfD\times BW)\;x\;MC{}^{-1}$$ (5)

Because As is carcinogenic, CR*lim* for arsenic was calculated according to Eq 6 [44].

$$CRlim=(ARL\times BW)\;x\;(MC{\;x\;CSF)}^{-1}$$ (6)

Where ARL is the maximum acceptable cancer risk level (10^−5^) [44].

Because daily consumption limit may be more conveniently expressed as allowable number of fish meals of a specific meal size over a given time [44], the maximum allowable fish consumption rates were converted to allowable number of fish meals per month (CR_mm_) according to Eq 7.

$$CRmm=(CRlim\;x\;Tap)\;x\;MS{}^{-1}$$ (7)

Where T_ap_ is time averaging period (365.25 days/12 months = 30.44 days per month) and MS denotes meal size for adults assumed 0.227 kg fish per meal [50]. Because the rate of fish consumption in Ghana is 0.0685 kg fish person per day [39] and less than the average fish meal size of 0.227 kg fish per meal [50], CR_mm_ was also separately calculated at 0.0685 kg fish meal. The calculation of CR_mm_ at the rate in which fish is eaten in Ghana enable us to determine the number of days that the fishes can be safely consumed in a month by the average Ghanaian if it is assumed that the 0.0685 kg fish that is eaten per person per day in Ghana are all contained in one meal [36, 37].

## Results

### Comparison of metal concentrations

**Fig 2:**
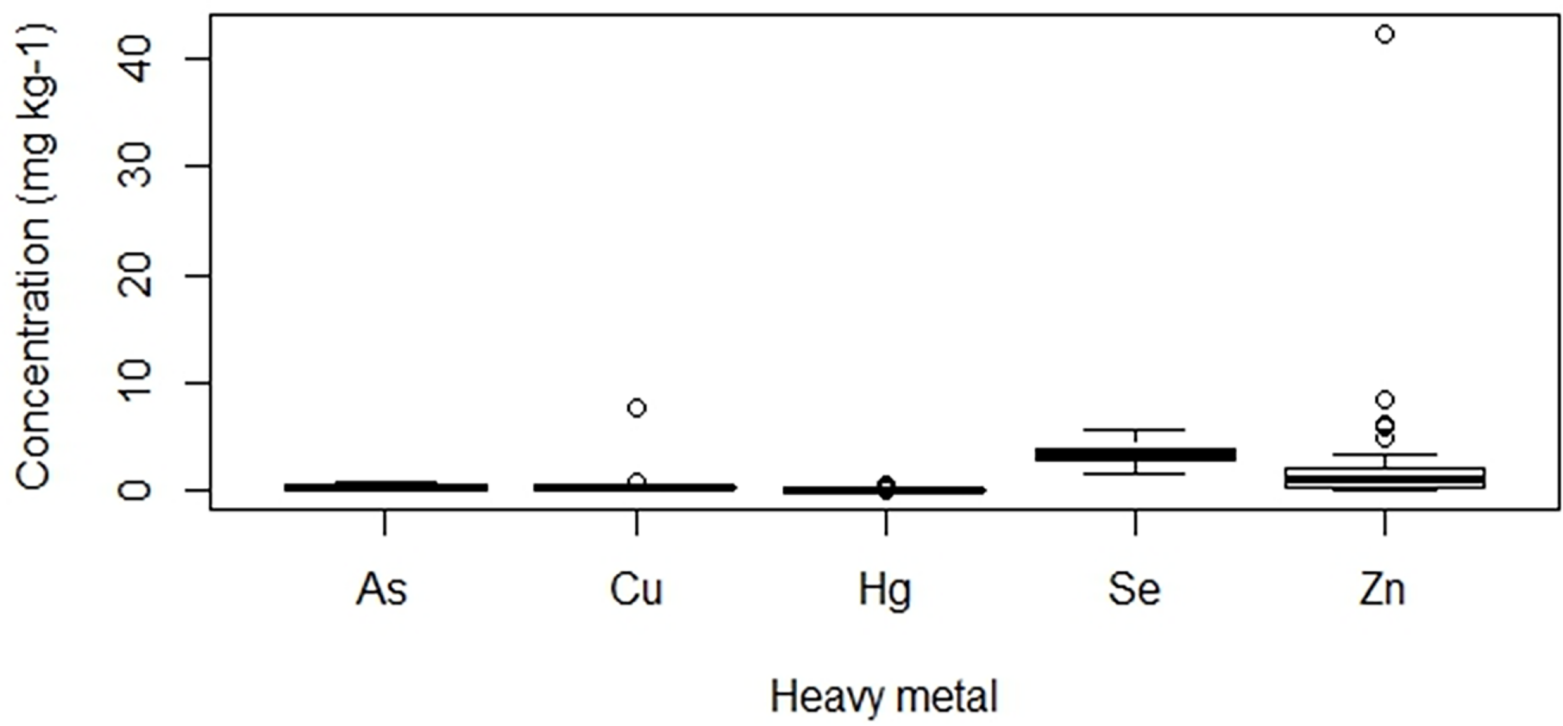
Trends in the concentration of heavy metals in the muscle tissues of *Chrysichthys nigrodigitatus* from the Weija Dam of the Densu River in Ghana. Note: Pb and Cd were below detection limits in C. nigrodigitatus. Note: Concentrations of As and Se, As and Zn, Hg and Se, as well as Hg and Zn were significantly different (Kruskal-Wallis Test with Fisher’s LSD post hoc, H_3,0.05_ = 118.779, p < 0.05).

**Fig 3:**
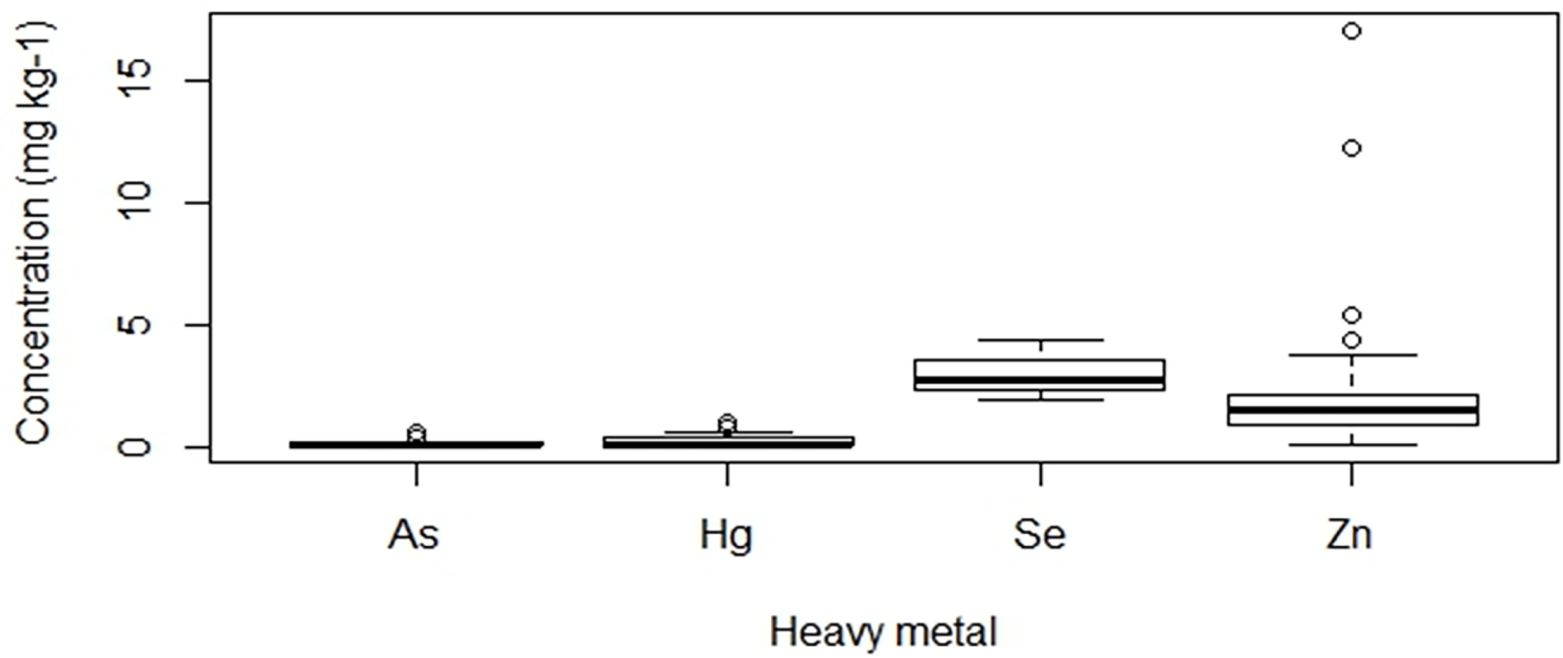
Trends in the concentration of heavy metals in the muscle tissues of *Brachydeuterus auritus* from the coastal waters of Ghana. Note: Pb, Cd and Cu were below detection limits in B. auritus. Also, pairwise comparison indicates concentration of all metals were significantly different except As and Hg (Kruskal-Wallis test with Fisher’s LSD post hoc, H_3,0.05_ = 129.618, p < 0.05.

The concentrations of heavy metals per kg muscle of *C*. *nigrodigitatus* (Fig 2) and *B*. *auritus* (Fig 3) indicated Se concentrations were the highest among the seven heavy metals. For both species of fish, Pb and Cd levels were below the detection limit of 0.2 and 0.4 mg kg^-1^ respectively while Cu with a detection limit of 0.42 was not detected in *B*. *auritus*. In *C*. *nigrodigitatus*, the concentration of metals (mg kg^-1^) followed the rank order Se (3.5) > Zn (2.34) > Cu (0.59) > As (0.37) Hg > (0.19) (Fig 2). Statistically, the concentrations of As and Se, As and Zn, Hg and Se, as well as Hg and Zn were significantly different (Kruskal-Wallis Test with Fisher’s LSD post hoc, H_3,0.05_ = 118.779, p < 0.05). With respect to *B*. *auritus*, metal concentrations (mg kg^-1^) occurred in the rank order Se (2.97) > Zn (2.28) > Hg (0.31) > As (0.21) (Fig 3). Except for Hg and As concentrations that were statistically similar, the concentration of the remaining metals in *B*. *auritus* were significantly different (Kruskal-Wallis test with Fisher’s LSD post hoc, H_3,0.05_ = 129.618, p < 0.05). Comparison of metal concentrations between the species indicated Se and As were significantly higher in the sampled muscles of *C*. *nigrodigitatus* than *B*. *auritus* while Hg was significantly higher in *B*. *auritus* (Mann–Whitney U test, p < 0.05) (Table 1). In contrast, Zn was statistically similar between the two species (Mann–Whitney U test, p < 0.0*5)* (Table 1).

**Table 1:** Comparison of heavy metals concentrations between the muscle tissues of *Chrysichthys nigrodigitatus* and *Brachydeuterus auritus* from the waters of Ghana. Note: *Mean ± SD with different letters in columns are significantly different (Mann–Whitney U test*, *p > 0*.*05)*.

	Concentrations (mg kg^-1^)				
	*Zn*	*Cu*	*Se*	*Hg*	*As*
	*Mean* *± SD*	*Mean* *± SD*	*Mean* *± SD*	*Mean* *± SD*	*Mean* *± SD*
*Chrysichthys nigrodigitatus*	2.34 ± 6.01**a**	0.59 ± 1.1	3.50 ± 0.91**b**	0.19 ± 0.13**b**	0.37 ± 0.24**b**
*Brachydeuterus auritus*	2.28 ± 2.90**a**	< 0.42	2.97 ± 0.71**a**	0.31 ± 0.27**a**	0.21 ± 0.12**a**
*P value*	z = 1.931		z = 2.979	z = 2.361	z = 3.378

### Metal concentrations in relation to permissible limits

**Fig 4:**
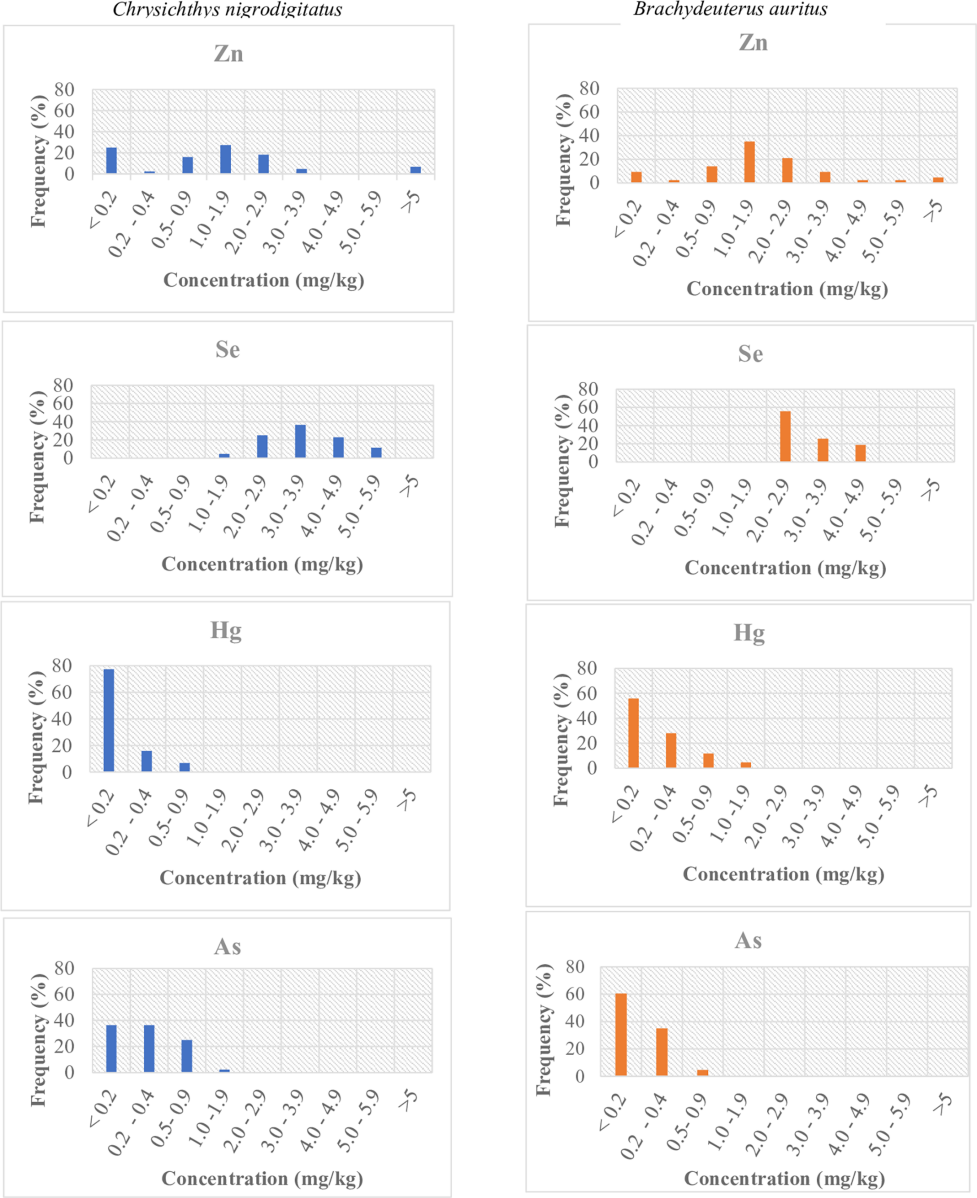
Frequency distribution of metal concentrations in the muscle tissues of *Chrysichthys nigrodigitatus* and *Brachydeuterus auritus* from the waters of Ghana. Note: Pb, Cd and Cu were excluded from Fig 1 because Pb and Cd were below detection in all samples while 96% of the C. nigrodigitatus and 100% of the B. auritus samples had Cu levels below detections.

Fig 4 shows the percentage frequency distribution of the concentrations of As, Hg, Se and Zn in the muscle of *C*. *nigrodigitatus* and *B*. *auritus*. Similar to the concentrations of Pb and Cd that were below detection, we did not include Cu concentrations in Fig 4 because, as much as 96% of the *C*. *nigrodigitatus* and 100% of the *B*. *auritus* samples had Cu levels below detections.

In relation to the toxic heavy metals, 93% of the sampled muscles of *C*. *nigrodigitatus* and 86% of *B*. *auritus* had Hg concentration below the maximum permissible limit of 0.5 mg kg^-1^. Of these analysed samples, 77% and 56% recorded less than 0.2 mg kg^-1^ Hg concentration respectively in *C*. *nigrodigitatus* and *B*. *auritus*. However about, 5% of *B*. *auritus* samples were found to have two to four-fold (1.0–1.9) mg kg^-1^ the maximum permissible Hg levels. Nonetheless, the corresponding Se content of these individuals were equally high ranging from (3.4–4.0) mg kg^-1^ with positive selenium health benefit values (HBV_Se_). For As however, 100% of the sample muscles of the *C*. *nigrodigitatus* and *B*. *auritus* had As concentrations lower than the 2 mg kg^-1^ maximum level permitted in fish.

In both species of fish, zinc and copper concentrations were respectively below the maximum permissible limit of 50 mg kg^-1^ and 40 mg kg^-1^ with only 7%, and 5% of samples recording above 5 mg kg^-1^ for *C*. *nigrodigitatus* and *B*. *auritus* respectively. In relation to Se, 95% and 100% of the *C*. *nigrodigitatus* and *B*. *auritus* respectively had Se concentration greater or equal to the standard of 2.0 mg kg^-1^.

### Estimated Daily / Weekly Fish Intake

Table 2 shows the human health risk parameters of heavy metals from the consumption of *C*. *nigrodigitatus* and *B*. *auritus*, from the waters of Ghana. For *C*. *nigrodigitatus*, the EDI values (mg kg^-1^ bw day^-1^) (Table 2) were in the order Hg < As < Cu < Zn < Se while the order As < Hg < Zn < Se was recorded for *B*. *auritus*. For both species of fish, the EDI of Zn and Cu were below the FAO/WHO PMTDI respective values of 0.8 and 0.5 mg kg^-1^ bodyweight indicating no serious health implications for consumers. For *C*. *nigrodigitatus*, the EDI of Hg and As respectively translates to EWI values of 1.4 and 2.8 ug kg^-1^ bodyweight a week. Similarly, the EDI of Hg and As for *B*. *auritus* respectively translates to EWI values of 2.1 and 1.4 ug kg^-1^ bodyweight which were lower than FAO/WHO PTWI respective values of 4 and 2.1 μg kg^-1^ bodyweight. Thus only As in *C*. *nigrodigitatus* had EWI values higher than the FAO/WHO PTWI of 2.1 μg kg^-1^ bodyweight and therefore raising concern.

**Table 2:** Human health risk assessment parameters of heavy metals from the consumption of *Chrysichthys nigrodigitatus* and *Brachydeuterus auritus* respectively from the fresh and coastal waters of Ghana.

Heavy metals	*Chrysichthys nigrodigitatus*						*Brachydeuterus auritus*					
	Estimated Daily Intake (mg kg^-1^ bw day^-1^)	Targeted Harzard Quotient		Maximum Safe consumption rate (kg fish day^-1^)	Maximum safe number of fish meals in a monthly basis		Estimated Daily Intake (mg kg^-1^ bw day^-1^)	Targeted Harzard Quotient		Maximum Safe consumption rate (kg fish day^-1^)	Maximum safe number of fish meals in a monthly basis	
		365 days a year	52 days a year		0.227 kg fish per meal	0.0658 kg fish per person per day		365 days a year	52 days a year		0.227 kg fish per meal	0.0658 kg fish per person per day
Zn	0.0023	0.008	0.001	8.974	1203	3988	0.0022	0.007	0.001	9.211	1235	4093
Cu	0.0006	0.014	0.002	4.746	636	2109	-	-	-	-	-	-
Se	0.0034	0.685	0.098	0.100	13	44	0.0029	0.581	0.083	0.118	16	52
Hg	0.0002	0.620	0.088	0.111	15	49	0.0003	1.011	0.144	0.068	9	30
As	0.0004	1.207	0.172	0.210	28	93	0.0002	0.685	0.098	0.370	50	165

### Estimation of Targeted Hazard Quotient and Cancer Risk of Arsenic

The THQ of *C*. *nigrodigitatus* and *B*. *auritus*, from the fresh and coastal waters of Ghana for individuals who consume the fish seven times a week and those who consume it once a week are presented in Table 2. The THQ values were generally below 1 except As in *C*. *nigrodigitatus* and Hg in *B*. *auritus* for individuals who eat the fish seven times a week indicating these individuals would likely experience some obvious adverse effects from the consumptions. With regard to the ameliorating effect of Se on Hg, calculated BHV_Se_ values for both *C*. *nigrodigitatus* (44.35 ± 11.55) and *B*. *auritus* (37.56 ± 8.77) were positive suggesting the health risks that might accompany Hg exposures would be negated.

In relation to the Risk of Cancer from the consumption of inorganic arsenic in fish, CR value for As in *C*. *nigrodigitatus* was 5.4 x 10^−10^ and that of *B*. *auritus* was 3.1 x 10^−10^. Since the values were up to 10^−10^, there is 1 chance in 10,000,000,000 of an individual developing cancer from the consumption of the fishes therefore indicating a negligible risk of cancer from the consumptions.

### Maximum allowable consumption rates and safe number of fish meals per month

The maximum safe consumption rates for *C*. *nigrodigitatus* and *B*. *auritus*, from the waters of Ghana are presented in Table 2. Also, Table 2 indicates the maximum number of times the fishes can be safely eaten in a month if the average meal of an adult contains 0.227 kg fish. For *C*. *nigrodigitatus*, CR*lim* ranged from 0.11 kg per day for Se to 8.97. kg per day for Zn. Similarly, CR*lim* for *B*. *auritus*, ranged from 0.07 kg per day for Se to 9.2 kg per day for Zn. In contrast, CR_mm_ values for *C*. *nigrodigitatus* ranged from 13 meals per month for Se to 1203 meals per month for Zn while *B*. *auritus* recorded CR_mm_ values from 9 meals per month for Hg to 1235 meals per month for Zn. On the basis of the rate at which fish is consumed in Ghana (0.0685 kg fish per person per day) [36, 37], CR_mm_ values for *C*. *nigrodigitatus* ranged from 44 meals per month for Se to 3988 meals per month for Zn while *B*. *auritus* recorded CR_mm_ values from 30 meals per month for Hg to 1235 meals per month for Zn, where it is assumed that the 0.0685 kg fish that is consumed per person per day constitute one safe meal (Table 2).

### Relationship between metal concentrations and fish morphometrics

**Fig 5:**
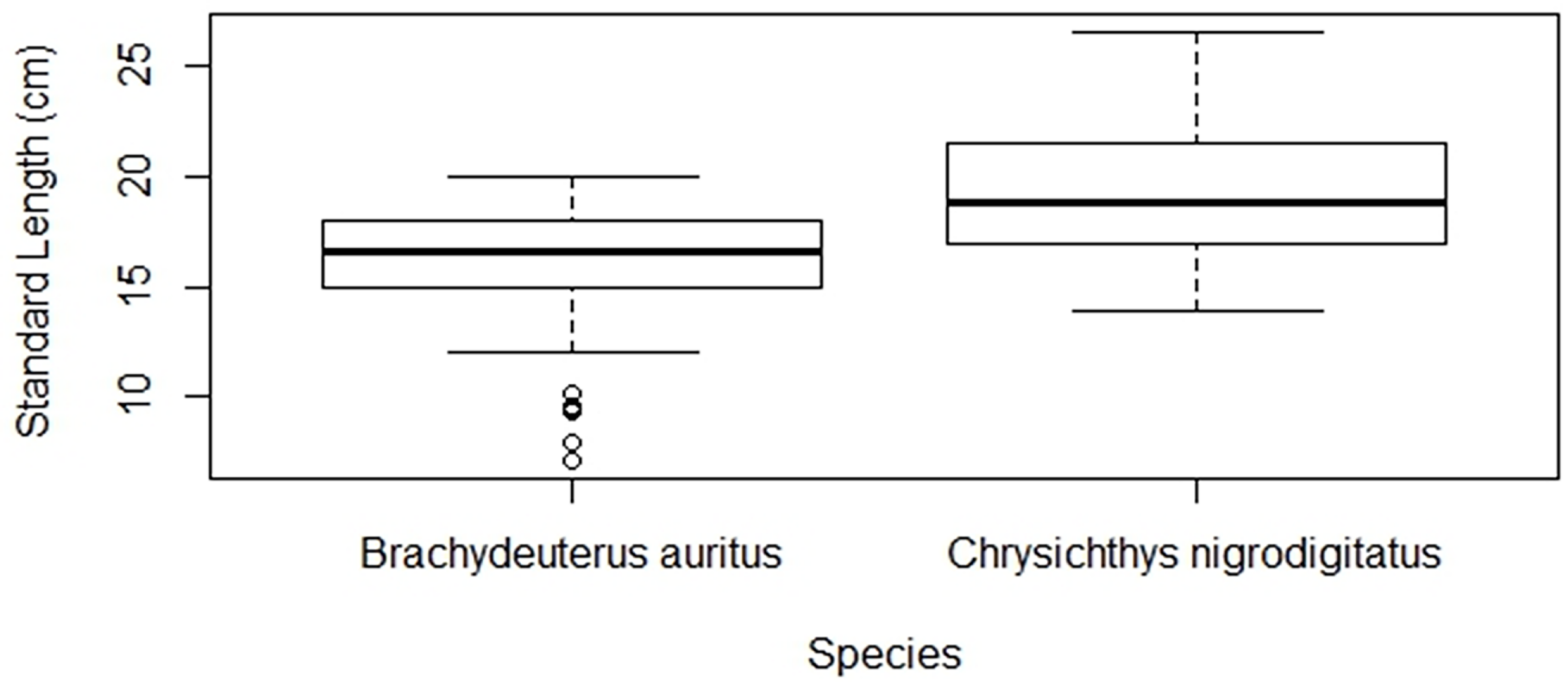
Standard length of *Chrysichthys nigrodigitatus* and *Brachydeuterus auritus* respectively from the fresh and coastal waters in Ghana.

**Fig 6:**
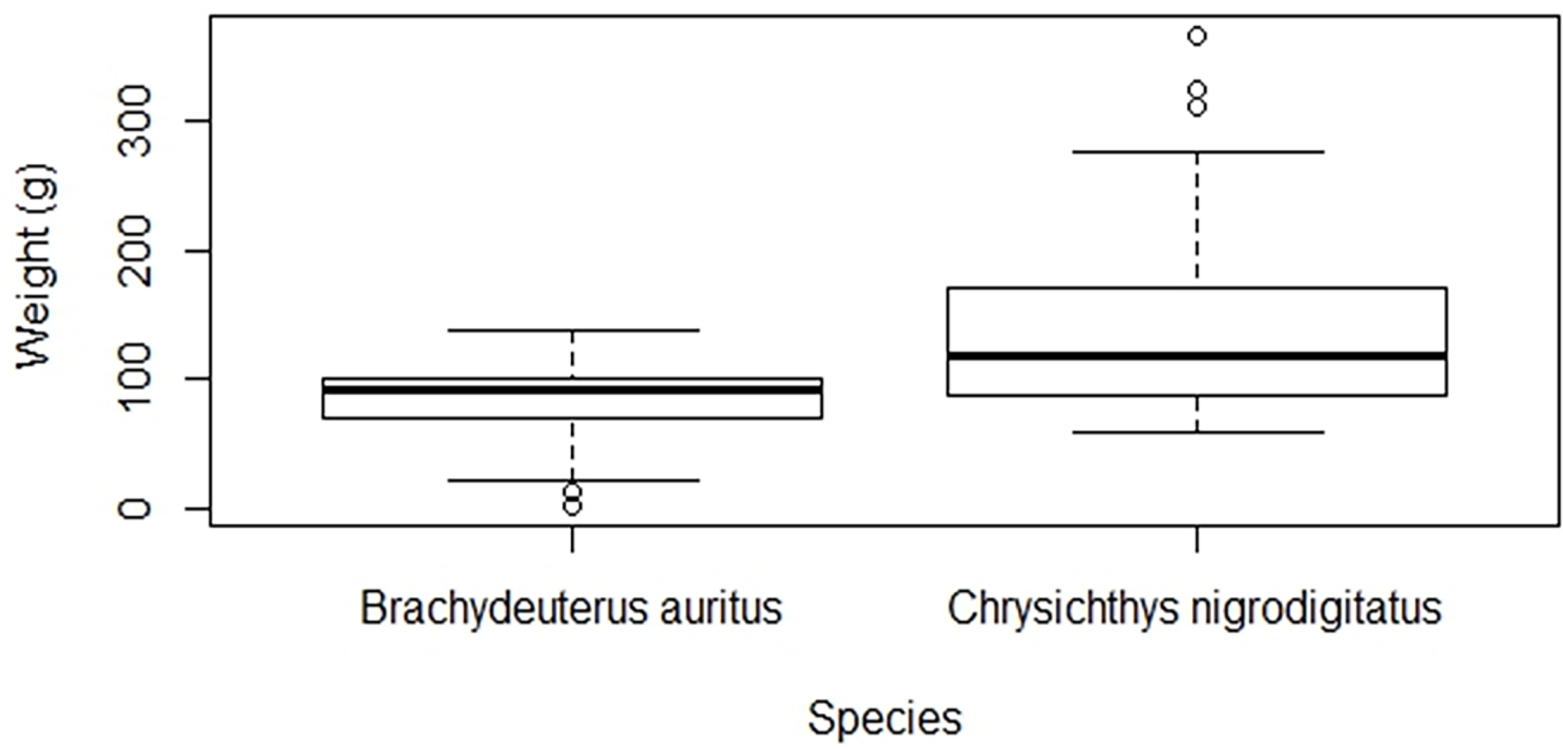
Weight of *Chrysichthys nigrodigitatus* and *Brachydeuterus auritus* respectively from the fresh and coastal waters in Ghana.

The Standard Length (cm) and Weight (g) of the two species of fish are presented in Figs 5 and 6. Mean weight of 145.0 ± 77.9 g and standard length of 19.6 ± 3.5 cm were recorded for the *C*. *nigrodigitatus* as against respective values of 84.0 ± 27.2 g and 16.0 ± 2.9 cm for *B*. *auritus*. The correlation analysis indicated that relationship between the concentrations of heavy metals in the muscles and weight of *C*. *nigrodigitatus* were insignificant (p > 0.05) for Hg (r = -0.05), As (r = 0.04), Se (r = 0.20), Zn (r = -0.14) and Cu (r = -0.28). In the same vein, the relationship between the metal concentration and weight of *B*. *auritus* was insignificant (p > 0.05) for Hg (r = 0.4), As (r = -0.13), Se (r = 0.78), Zn (r = - 1.0) and Cu (r = 0.14). Further, the relationship between the Standard Length and metal levels in *C*. *nigrodigitatus* was insignificant (p > 0.05) for Hg (r = 0.03), As (r = 0.14), Se (r = 0.09), Zn (r = - 0.10), and Cu (r = 0.06) just as that of *B*. *auritus* that was insignificant for Hg (r = 0.17), As (r = 0.03), Se (r = 0.14), Zn (r = 0.47), and Cu (r = 0.54).

## Discussion

### Metal levels

The fear of heavy metal poisoning among consumers of Ghana’s fisheries resource appears to be on the increase as a consequence of several anthropogenic processes; most prominent one being the activities of artisanal mining of gold and refinery along the major rivers and watershed areas in the country. The goal of this study was to evaluate the potential of heavy metal toxicity from the consumption of two valuable commercial species of fish from the fresh and coastal waters of Ghana. The results indicated that the rank orders of heavy metals in both *C*. *nigrodigitatus* and *B*. *auritus* were similar with the concentrations of the essential metals (Se, Zn and Cu) generally higher than the toxic metals (Hg and As). In a snap shot survey, a higher levels of Hg was recorded in *C*. *nigrodigitatus* from the Densu Delta section of the Densu River than reported in this current study [5]. This is not surprising because downstream dam fishes are known to bioaccumulate more Hg than upstream fishes [51]. Thus, the higher levels of Hg observed in *C*. *nigrodigitatus* in the Densu Delta [5] compared to the Weija Dam was expected.

In the current study, comparison of metal concentrations between the *C*. *nigrodigitatus* and *B*. *auritus* indicated that As was higher in *C*. *nigrodigitatus* than *B*. *auritus* whilst Hg was higher in *B*. *auritus* than in *C*. *nigrodigitatus*. Because some heavy metals such as As accumulate in large quantities in the sediments on the bed of water bodies, their concentrations are generally higher in demersal aquatic species than pelagic organisms [52]. Our findings with respect to arsenic levels in the fish species was thus not surprising. Also, because the anthropogenic sources of heavy metal pollution (especially the use of mercury in the refinery of gold) are closer to the freshwater environment than the marine environment, it was expected that the concentrations of Hg per kg of muscle in *C*. *nigrodigitatus* would be higher than *B*. *auritus*. However, our data indicated the opposite and therefore suggesting that *B*. *auritus* readily bioaccumulates Hg as a result of its carnivorous feeding habit as against the omnivorous feeding habits of *C*. *nigrodigitatus* [10, 53]. It is known that piscivorous fish species generally have higher mercury load than non-piscivorous fish species [54]. Further, because *B*. *auritus* are known to forage over large distances [21], their heavy metal levels might also integrate contamination from diverse areas unlike the *C*. *nigrodigitatus* that were restricted to the Densu River. Nevertheless, the result significantly supports the fact that the contamination of freshwater resources does not immediately translate to bioaccumulation to toxic levels in primary and secondary consumers of the contaminated water but may pose a problem with species high up the food chain.

### Metal concentrations in relation to permissible limits

Given that both As and Hg concentrations in the two species were largely below the maximum permissible limits [29, 30, 31], there appear to be minimal risk of As and Hg toxicity associated with the consumption of *C*. *nigrodigitatus* from the Densu River and *B*. *auritus* from the coastal waters of Ghana. Similar levels of safety from the consumption of fish have been reported in China [55, 56].

Regarding the essential heavy metals, although they are necessary for human growth and metabolism, they have high toxicity effects on the body at high tissue concentrations [3]. Our data indicated that Zn and Cu were respectively below the maximum permissible limit of 50 mg kg^-1^ and 40 mg kg^-1^ [32, 33, 34] and therefore indicating no perceived risk to consumers. Given the biological significance of these trace elements (Zn and Cu) in metabolism (e.g. as co-factors of enzymes), the levels found in the two species of fish makes the fishes important sources of these micronutrients, thus highlighting the significance of their nutritional values for consumers.

Selenium, another trace element of major importance in human health and nutrition is known to counteract mercury toxicity through the high affinity binding between the selenol groups of selenoproteins and Hg, as well as the antioxidative action of the selenoproteins on the reactive oxygen species induced by Hg [57, 58, 59]. Although there is no current FAO/WHO maximum permissible limit for Se, our data shows concentrations in the fishes were generally greater or equal to the standard of 2.0 mg kg-1 [35]. In respect of its mercuric counteracting effects, our data indicated that the HBV_Se_ of both *C*. *nigrodigitatus* (44.35 ± 11.55) and *B*. *auritus* (37.56 ± 8.77) were positive and compared favourably with those reported for fishes in some other regions of the world [38], suggesting that the two species pose no risk of mercury toxicity to consumers but are rich source of Se.

### Human health risk assessment

The risk of toxicity of a metal is determined by the concentration of the metal in a source and the quantity of the source that is consumed [60]. Tolerable daily and weekly intake values of contaminants are therefore determined to balance the risk of consumption with the nutritional benefits such as proteins, phosphorus, omega-3 fatty acids, calcium, minerals and some vitamins that are associated with fish consumption. Our data indicated that EDI and EWI values of all the metals were lower than the FAO/WHO PTWI and PMTDI except for arsenic in *C*. *nigrodigitatus*. Thus, except for As in *C*. *nigrodigitatus*, the recorded EDI values were generally not concerning. While the As levels in *C*. *nigrodigitatus* might be concerning, As in fish generally exists in non-toxic organic forms such that the toxic inorganic As content of fish is generally less than 10% [45]. However, taken together with the calculation of THQ based on 3% of the total As recorded in this study, which produced a THQ value that was greater than one (1) for individuals who eat *C*. *nigrodigitatus* seven times a week, the possibility of the occurrence of adverse effects associated with As from the regular consumption of *C*. *nigrodigitatus* from the Weija Dam of the Densu River may be real. Although adverse effects may be associated with the regular consumption of *C*. *nigrodigitatus* from the Weija Dam of the Densu River as a result of As load, the effect excludes the risk of cancer. The Risk of Cancer from the consumption of inorganic As in *C*. *nigrodigitatus* was 5.4 x 10^−10^ indicating a negligible risk of 1 chance of cancer development in 10,000,000,000 individual that are regularly exposed to the fishes.

In relation to, *B*. *auritus*, Hg produced a THQ value marginally greater than one (> 1) for individuals who consume *B*. *auritus* on daily basis suggesting a marginal possibility of the observation of adverse effect resulting from the Hg load. On the bases of the Se content of the fish however, no mercury toxicity health risk can be ascribed to the consumption of this fish because of the positive HBV_Se_ values recorded.

In terms of the maximum allowable fish consumption rates, As recorded a maximum safe consumption rate of 0.21 mg *C*. *nigrodigitatus* per day while Hg recorded 0.07 mg *B*. *auritus* per day. These values translated into not more than 28 *C*. *nigrodigitatus* and nine (9) *B*. *auritus* meals in a month at the average fish meal size of 0.227 kg fish per meal for an adult. That notwithstanding, because only 0.0685 kg of fish is consumed per person per day in Ghana [36,37] he quantity of fish meals in a month to attain these maxima translates into 93 for *C*. *nigrodigitatus* and 30 for *B*. *auritus* meals respectively (Table 2). These suggests, a maximum of three *C*. *nigrodigitatus* meals may be consumed per day to avoid As toxicity while one *B*. *auritus* meals may be consumed per day in the absence of Se in the fish or other components of the meal. Therefore at the rate of 0.0685 kg of fish per person per day in Ghana, the consumption of the two species of fish would essentially be of little or no consequence to consumers.

It is worth noting that heavy metals such as Hg are disproportionately distributed in organs such as the skin, kidneys, skeletal muscles and bones [61]. Indeed, significant differences were reported in the concentration of heavy metals between the skin and muscles of *Scomberomorus guttatus* but not in *Otolithes ruber* [62]. As skeletal muscle constitutes the most significant component of fish meat, the health risk assessments in this current study were based on the metal contents of the skeletal muscle tissues only and therefore caution must be exercised in the use of the figures.

## Conclusions

This current study established that the rank order of heavy metals in *C*. *nigrodigitatus* and *B*. *auritus* was similar with the concentrations of the essential metals generally higher than the toxic ones. Comparatively, Zn and Se concentrations were similar in the two species of fish but As was higher in *C*. *nigrodigitatus* whilst Hg was higher in *B*. *auritus*. The concentrations of Zn, Cu, As and Hg were below the maximum permissible limits in fish and except for As in *C*. *nigrodigitatus*, EDI / EWI values were generally less than FAO/WHO PTWI and PMTDI values indicating minimal toxicity risk to consumers. Calculated THQ values for As in *C*. *nigrodigitatus* and Hg in *B*. *auritus* for individuals consuming the fishes on daily basis were greater than 1 indicating the possibility of the occurrence of adverse effect. However, for both species of fish, cancer risk was negligible with a probability of one (1) in 10,000,000,000 individual. Also, calculated BHV_Se_ values for both *C*. *nigrodigitatus* and *B*. *auritus* were positive suggesting the health risks that might accompany Hg exposures would be negated. With a maximum safe consumption rate of 0.21 mg *C*. *nigrodigitatus* per day for As, and 0.07 mg *B*. *auritus* per day of Hg, not more than 24 *C*. *nigrodigitatus* meals per month and nine (9) *B*. *auritus* meals in a month were recommended at an adult fish meal size of 0.227kg fish per meal. Because fish is generally consumed at a far less quantity per person in Ghana, the quantity of fish involved in the safe number of meals per month are barely eaten at the rate in which fish is consumed in Ghana. Therefore, the consumption of the two species of fish would essentially be of little or no consequence to consumers at its current rate of consumption.
